# The impact of curative conversion therapy aimed at a cancer‐free state in patients with hepatocellular carcinoma treated with atezolizumab plus bevacizumab

**DOI:** 10.1002/cam4.5931

**Published:** 2023-04-16

**Authors:** Shigeo Shimose, Hideki Iwamoto, Tomotake Shirono, Masatoshi Tanaka, Takashi Niizeki, Masahiko Kajiwara, Satoshi Itano, Yoichi Yano, Satoru Matsugaki, Etsuko Moriyama, Yu Noda, Masahito Nakano, Ryoko Kuromatsu, Hironori Koga, Takumi Kawaguchi

**Affiliations:** ^1^ Division of Gastroenterology, Department of Medicine Kurume University School of Medicine Kurume Fukuoka Japan; ^2^ Iwamoto Internal Medical Clinic Kitakyusyu Japan; ^3^ Yokokura Hospital, Clinical Research Center Miyama Fukuoka Japan; ^4^ Department of Gastroenterology Chikugo City Hospital Chikugo Japan; ^5^ Department of Gastroenterology Kurume Central Hospital Kurume Japan; ^6^ Division of Gastroenterology, Department of Medicine Japan Community Health Care Organization, Saga Central Hospital Saga Japan; ^7^ Department of Gastroenterology Tobata Kyoritsu Hospital Kitakyusyu Japan

**Keywords:** atezolizumab plus bevacizumab, conversion therapy, hepatocellular carcinoma

## Abstract

**Background and Aims:**

We aimed to validate the predictive factors for tumor response and the prognostic impact of conversion therapy aimed at cancer‐ and drug‐free states in patients with unresectable hepatocellular carcinoma (u‐HCC) undergoing atezolizumab plus bevacizumab (Atez/Bev) therapy.

**Methods:**

This retrospective study enrolled 156 patients who were Child‐Pugh class A with u‐HCC treated using Atez/Beva. The profile of objective response was investigated using decision‐tree analysis. Progression‐free, recurrence‐free, and overall survival were assessed.

**Results:**

The progression‐free and overall survival were 6.1 and 18.0 months, respectively. Objective response and disease control rates were 32.0% and 84.0%, respectively. Decision‐tree analysis revealed that neutrophil‐to‐lymphocyte ratio (NLR) <3, modified albumin‐bilirubin grade (m‐ALBI) 1 or 2a, and age < 75 were sequential splitting variables for the objective response, respectively. In the multivariate analysis, NLR <3 and m‐ALBI grade 1 or 2a were identified as predictive factors for objective response. We successfully achieved eligibility for conversion therapy in 17 patients after Atez/Bev therapy significant response. Following conversion therapy, the curative therapy group, including surgical resection or radiofrequency ablation (RFA), had significantly higher recurrence‐free survival than did the transcatheter arterial chemoembolization (TACE) and Atez/Bev discontinuation (surgical resection or RFA; not reached vs. TACE; 5.3 months, *p* = 0.008, Atez/Bev discontinuation; 3.9 months, *p* = 0.048, respectively) groups.

**Conclusions:**

NLR <3 and m‐ALBI grade 1 or 2a were predictive factors for conversion therapy, leading to cancer‐ and drug‐free states in patients with u‐HCC undergoing Atez/Bev therapy. Moreover, surgery or RFA may be suitable for conversion therapy for cancer‐free status.

## Graphical abstract

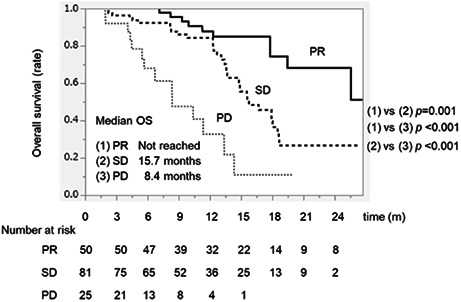

## 1 INTRODUCTION

Hepatocellular carcinoma (HCC), the major malignant primary liver cancer, is the third leading cause of cancer‐related deaths worldwide.^1^, ^2^ Many patients with unresectable HCC (u‐HCC) receive systemic therapy because HCC is often diagnosed at an advanced stage.^3^, ^4^ Thus, several molecular targeted agents for treating u‐HCC have been developed, such as sorafenib,^5^ lenvatinib,^6^ ramucirumab,^7^ and cabozantinib.^8^ Moreover, immune checkpoint inhibitors have recently revolutionized the treatment strategy for u‐HCC.

Atezolizumab plus bevacizumab (Atez/Bev), a combination of the atezolizumab (anti‐programmed death ligand‐1 inhibitor) and bevacizumab (vascular endothelial growth factor inhibitor), was approved for the first‐line systemic therapy for patients with u‐HCC based on the IMbrave150 trial's finding.^9^ Atez/Bev treatment improved progression‐free survival (PFS) and overall survival (OS) compared to sorafenib, and it is expected to shrink tumors more than the previously used systemic therapy.^9^, ^10^, ^11^

Currently, the treatment strategy for u‐HCC is sequential therapy with systemic therapy.^12^, ^13^, ^14^ However, cancer‐free conversion therapy, such as surgery, radiofrequency ablation (RFA), or curative transarterial chemoembolization, can be performed if adequate tumor reduction is achieved with Atez/Bev therapy.^15^ Several reports have discovered that OS was prolonged in patients who received liver resection or RFA as conversion therapy for colorectal cancer liver metastasis after tumor reduction with chemotherapy.^16^, ^17^, ^18^, ^19^ Thus, establishing predictors of tumor shrinkage is important. Previous studies reported that neutrophil‐to‐lymphocyte ratio (NLR), albumin‐bilirubin (ALBI) grade,^20^, ^21^ neo‐Glasgow prognostic scores,^22^ and prognostic nutritional index^23^ were the predictive factors associated with therapeutic effect or OS in Atez/Bev treatment for HCC. However, the predictive factors for the response to Atez/Bev treatment have not yet been established. Moreover, few studies have reported the impact of conversion therapy aimed at cancer‐ and drug‐free status after Atez/Bev treatment, and the associated outcomes are unknown.

Therefore, in this study, we aimed to investigate the predictive factors for tumor response in u‐HCC patients treated with Atez/Bev. Additionally, we aimed to evaluate the impact of conversion therapy aimed at cancer‐ and drug‐free states after an effective response to Atez/Bev treatment.

## 2 METHODS

### 2.1 Patients

Between 2020 and October 31, 2022, this multicenter retrospective cohort study enrolled 177 consecutive u‐HCC patients treated with Atez/Bev at seven Japanese institutions. The following eligibility criteria were used: (i) age > 18 years; (ii) Eastern Cooperative Oncology Group performance status (PS) <2; and (iii) clinical data were available and it could follow‐up until study cessation (December 31, 2022) or death. Exclusion criteria were as follows: (i) Child‐Pugh class B or C; (ii) history of autoimmune disease; and (iii) active esophageal varices. Thus, 21 patients were excluded. Finally, this study enrolled 156 patients (Figure S1).

This research was conducted according to the Helsinki Declaration and approved by the Ethics Committee of the Kurume University School of Medicine (approval code: 20183). An opt‐out approach was used to obtain informed consent from patients.

### 2.2 Confirmation of HCC

HCC confirmation was based on hypervascularization in the arterial phase and washout in the portal venous phase or delayed phase on CT or MRI examination. Additionally, in MRI, focal areas with a suspicious hypointense signal in the hepatobiliary phase were used to detect HCC. For the cutoff of tumor marker level, AFP ≥7 was considered positive in this study. HCC was diagnosed using a combination of serum markers and imaging following recommendations of The Japan Society of Hepatology.^24^

### 2.3 Treatment protocol

The patient was administrated Atez/Bev according to the recommended dosage (1200 mg of Atez and 15 mg/kg of Bev intravenously every 3 weeks). The clinical guidelines for Atez/Bev created by the manufacturer were used for the discontinuation of each component agent if a treatment‐related adverse event occurred. Atezolizumab therapy was interrupted when any unacceptable grade 2 or over grade 3 immune‐related AEs occurred such as liver injury, skin disorder, and drug‐induced pneumonia.^9^, ^25^ These patients received bevacizumab monotherapy. Bevacizumab was interrupted when any unacceptable grade 2 or over grade 3 bevacizumab‐related AEs occurred such as proteinuria and bleeding.^26^ These patients received atezolizumab monotherapy. Patients received the treatment until the development of unacceptable adverse events (AEs) or tumor progression. Atez/Bev could be continued even beyond tumor progression if clinical benefit is still observed; among patients who refused other systemic treatments, Atez/Bev was continued if disease progression was slow and adverse events of Atez/Bev treatment were acceptable.

### 2.4 Assessment of therapeutic response and safety

The Response Evaluation Criteria In Solid Tumors version 1.1 (RECIST v1.1).^27^ was used to evaluate therapeutic response using CT/MRI 6 weeks after the initiation of Atez/Bev. The Common Terminology Criteria for Adverse Events version 5.0 was adopted to assess AEs every 3 weeks until study cessation or death.^28^

### 2.5 Definition of eligibility for conversion therapy after Atez/Bev and additional conversion therapy

We defined eligibility for conversion therapy as follows: (1) no viable lesion on CT or MRI (such as necrotic cases) scans and range within normal values for tumor markers; AFP was the independent risk factor for early recurrence after surgical resection for HCC,^29^, ^30^ and (2) condition in which additional treatment was expected to achieve cancer‐ and drug‐free statuses. We recommended all patients who fulfilled the eligibility criteria for conversion therapy to achieve cancer‐ and drug‐free state, and the optimal treatment for conversion therapy for each patient was determined by discussions among hepatologists, radiologists, and surgeons following the Japanese practice guidelines for HCC.^24^

### 2.6 The decision‐tree algorithm

A decision‐tree algorithm was constructed to reveal the profiles' relationship with conversion therapy and objective response (OR) following the instructions provided with the R software package as described previously.^14^, ^31^ The profiles were identified by data‐mining methods.

### 2.7 Statistical analysis

All provided data were described as medians (ranges). Between‐group comparisons were carried out using the Mann–Whitney U test, the Kruskal–Wallis test, and a nonparametric analysis of variance. If the one‐way analysis of variance was significant, differences between individual groups were analyzed using the Fisher least significant difference test. Evaluation of PFS, recurrence‐free survival (RFS), and OS was carried out using the Kaplan–Meier method, and statistical differences were evaluated with the log‐rank test or Bonferroni method. A decision‐tree analysis was carried out to investigate the factors of conversion therapy and objective response (OR), as previously described.^14^ Statistical significance difference was defined as *p* < 0.05. Statistical analyses were performed using JMP software (JMP Pro version 15, SAS Institute Inc.).

## 3 RESULTS

### 3.1 Clinical characteristics

The characteristics of the 156 patients are listed in Table 1. The median age was 73 (37–93) years, and there were 35 (22.4%) females and 121 (77.6%) males. There were 49 (31.4%), 57 (36.5%), and 50 (32.1%) patients with modified ALBI (m‐ALBI) grade 1, m‐ALBI grade 2a, and m‐ALBI grade 2b, respectively. The median observation time was 11.2 months (2.1–26.0). Seventeen patients were eligible for conversion therapy after Atez/Bev significant response.

**TABLE 1:** Patient characteristics.

Characteristic	All patients
*N*	156
Age (years old)	73 (37–93)
Sex (female/male)	35/121
ECOG PS (0/1)	128/28
Body mass index (kg/m^2^)	22.9 (15.4–35.2)
Etiology (HBV/HCV/Non B, C)	25/70/61
Albumin (g/dL)	3.7 (2.81–5.0)
Total bilirubin (mg/dL)	0.8 (0.3–2.1)
ALBI score (median [range])	−2.41 (−3.50 to −1.55)
m‐ALBI grade (1/2a/2b)	49/57/50
White blood cell (/μL)	4650 (1900‐9800)
Neutrophil lymphocyte ratio	2.50 (0.65–11.1)
BCLC stage (B/C)	78/78
Tumor diameter (mm)	32.5 (10–136)
Number of tumors < 5/ ≥ 5	46/110
Macrovascular invasion (No/Yes)	132/24
Extrahepatic spread (No/Yes)	97/59
AFP (ng/mL)	37.3 (1.2–284,543)
Treatment line (1st/2nd/3rd/4th)	95/49/8/4
Follow‐up duration (months)	11.2 (2.1–26.0)
Eligible for conversion therapy (No/Yes)	139/17

*Note*: Data are expressed as median (range), or number.

Abbreviations: AFP, α‐fetoprotein; BCLC, Barcelona Clinic Liver Cancer; ECOG PS, Eastern Cooperative Oncology Group Performance Status; HBV, hepatitis B virus; HCV, hepatitis C virus; m‐ALBI, modified albumin‐bilirubin.

### 3.2 Overall therapeutic outcomes of Atez/Bev

The median PFS and median survival time (MST) were 6.1 and 18.0 months (Figure S2A,B). In the radiological best response rates for complete response (CR), partial response (PR), stable disease (SD), and progressive disease (PD) were 0.0%, 32.0%, 52.0%, and 16.0%, respectively. The overall response rate (ORR) was 32.0%, and the disease control rate (DCR) was 84.0% (Table S1).

### 3.3 Decision tree associated with possible conversion therapy

**FIGURE 1:**
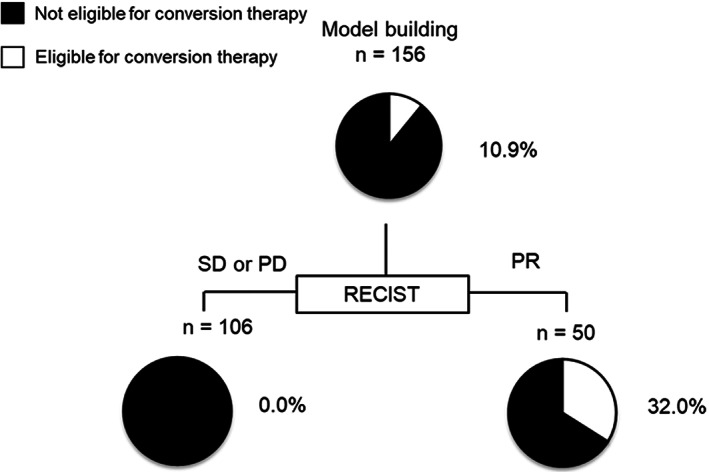
Profiles associated with possible conversion therapy in patients with HCC treated with Atez/Bev. Eligibility for conversion therapy. The pie graphs indicate the percentage of possible conversion therapy (white)/impossible conversion therapy (black) in each group. HCC, hepatocellular carcinoma.

In the present study, the conversion therapy rate in all participants was 10.9% (Figure 1). A decision‐tree analysis was performed to determine the profiles related to conversion therapy. RECIST was detected as the first splitting factor for the conversion therapy rate. The conversion therapy rate was 0.0% in patients with SD or PD treated with Atez/Bev; however, the conversion therapy rate was 32% in patients with PR treated with Atez/Bev.

### 3.4 Overall survival according to treatment response by RECIST

**FIGURE 2:**
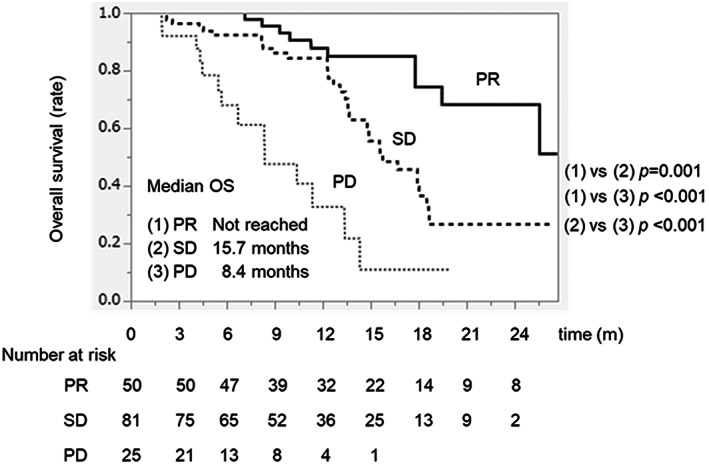
Overall survival duration in patients with HCC treated with Atez/Bev according to treatment response by RECIST. The black, dotted, and thin line indicates the PR, SD, and PD group, respectively. Atez/Bev, atezolizumab plus bevacizumab; CR, complete response; HCC, hepatocellular carcinoma; PD, progressive disease; PR, partial response; RECIST, response evaluation criteria in solid tumors; SD, stable disease.

The MST was not reached, 15.7 months, and 8.4 months among patients who achieved PR, SD, and PD, respectively (Figure 2). The PR group had a significantly higher MST than the SD group (not reached vs. 15.7 months, *p* = 0.001). In addition, the SD group had a significantly higher MST than the PD group (MST: 15.7 months vs. 8.4 months, *p* < 0.001).

### 3.5 Decision‐tree for OR

**FIGURE 3:**
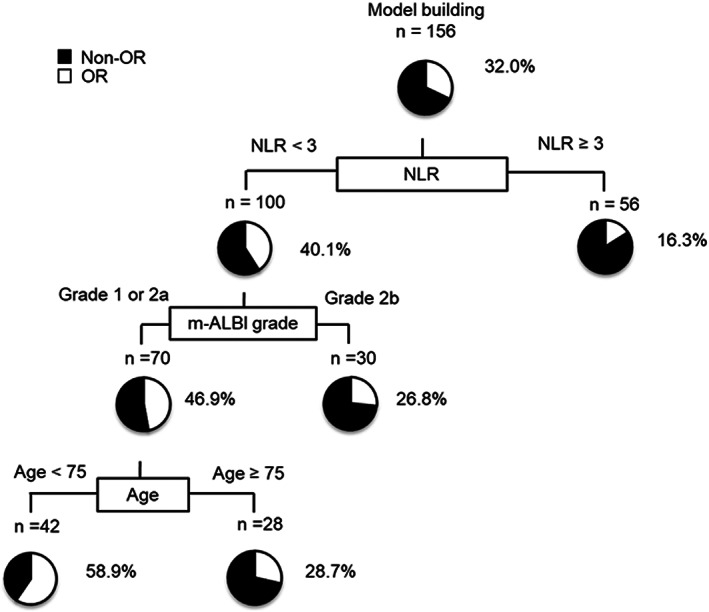
Profiles related to OR in HCC patients treated with Atez/Bev. Decision‐tree algorithm for OR. The pie graphs indicate the percentage of OR (white)/non‐OR (black) in each group. Atez/Bev, atezolizumab plus bevacizumab; HCC, hepatocellular carcinoma; OR, objective response.

In the present study, the OR rate in all participants was 32.0% (Figure 3). A decision‐tree analysis was conducted to determine the profiles related to OR. NLR was detected as the first splitting variable for OR. The OR rate was 40.1% in patients with NLR <3, while it was only 16.3% in patients with NLR ≥3. In patients with NLR <3, the 2nd and 3rd splitting factors were m‐ALBI grade and age. The OR rate was 58.9% in patients with NLR <3, m‐ALBI grade 1 or 2a, and age < 75.

### 3.6 Multivariate analyses of variables associated with OR and OS

NLR and m‐ALBI grade were identified as independent variables for OR (Table 2), while NLR, AFP, and m‐ALBI grade were identified as independent variables for OS (Table 3).

**TABLE 2:** Univariate and multivariate analyses of factors for OR.

	Univariate analysis	Multivariate analysis		
	*p*‐value	HR	95% CI	*p*‐value
Age, <75 vs ≥75	0.046			
Sex, male vs female	0.928			
Etiology HBV, vs HCV, versus Non B, C	0.645			
m‐ALBI grade 1 or 2a vs 2b	0.012	0.434	0.207–0.914	0.025
Maximum tumor diameter <30/≥30 (mm)	0.221			
Number of tumors <5/≥5	0.113			
Macrovascular invasion (Yes/No)	0.884			
Extrahepatic spread (Yes/No)	0.744			
AFP, <400 vs. ≥400 ng/mL	0.732			
NLR, <3 vs. ≥3 ng/mL	0.001	0.287	0.125–0.662	0.001
1st‐line vs later line	0.585			

Abbreviations: AFP, α‐fetoprotein; ALBI grade, Albumin‐bilirubin grade; HBV, hepatitis B virus; HCV, hepatitis C virus; NLR, neutrophil lymphocyte ratio; OS, overall survival.

**TABLE 3:** Univariate and multivariate analyses of factors for OS.

	Univariate analysis	Multivariate analysis		
	*p*‐value	HR	95% CI	*p*‐value
Age, <75 vs ≥75	0.986			
Sex, male vs female	0.201			
Etiology HBV, vs HCV, vs Non B, C	0.254			
m‐ALBI grade 1 or 2a vs 2b	0.038	0.589	0.371–0.982	0.042
Maximum tumor diameter <30/≥30 (mm)	0.101			
Number of tumors < 5/≥ 5	0.105			
Macrovascular invasion (Yes/No)	0.529			
Extrahepatic spread (Yes/No)	0.223			
AFP, <400 vs. ≥400 ng/mL	0.007	0.374	0.214–0.653	0.001
NLR, <3 vs. ≥3 ng/mL	0.001	0.207	0.117–0.366	<0.001
1st‐line vs later line	0.964			

Abbreviations: AFP, α‐fetoprotein; ALBI grade, Albumin‐bilirubin grade; HBV, hepatitis B virus; HCV, hepatitis C virus; NLR, neutrophil lymphocyte ratio; OS, overall survival.

### 3.7 Patient characteristics with NLR <3 and ≥3

The characteristics of patients in the NLR <3 and ≥3 groups are summarized in Table S2. There were no significant differences in age, sex, m‐ALBI grade, extrahepatic spread, macrovascular invasion, AFP, or treatment lines between the two groups. However, tumor size and tumor number were significantly more frequent in the NLR >3 group than those in the NLR <3 group.

### 3.8 The eligibility for conversion therapy after Atez/Bev therapy

The proportion of patients who were eligible for conversion therapy was 10.9% (17/156). Five, three, and three patients underwent surgery, RFA, and transarterial chemoembolization, respectively, as a treatment for conversion therapy. The prevalence of curative therapy as surgery and RFA was 47.1% (Table 4). All six patients for whom Atez/Bev treatment was discontinued belonged to the no viable lesion on imaging category and tumor markers within normal ranges; however, it was impossible to predict pathological CR among these patients, and so we recommended these patients for additional treatment aimed at a cancer‐free state because of the risk of developing adverse events due to continued Atez/Bev treatment. However, these patients requested to discontinue Atez/Bev treatment, and we chose the strategy of discontinuing Atez/Bev treatment after Atez/Bev receipt for 3 months at their request. The median time from the start of Atez/Bev therapy to conversion therapy was 292 days (range 84–616 days).

**TABLE 4:** Conversion therapy after Atez/Beva therapy.

Variables	*N* = 156
Conversion therapy (Yes/No)	17/139
Conversion rate	10.9% (17/156)
Surgery	5
RFA	3
TACE	3
Atez/Bev discontinuation	6
Curative therapy (Surgery or RFA)	47.1% (8/17)
Non‐curative therapy	52.9% (9/17)

Abbreviations: Atez/Bev, atezolizumab plus Bevacizumab; RFA, radiofrequency ablation; TACE: transarterial chemoembolization.

### 3.9 OS with and without conversion therapy

OS in the conversion therapy group was significantly higher than that in the non‐conversion therapy group (MST: not reached vs. 15.6 months, *p* < 0.001) (Figure S3).

### 3.10 Patient characteristics at the time of receiving conversion therapy with surgery or RFA and TACE

The characteristics of patients at the time of receipt of conversion therapy are listed in Table S3. There were no significant differences in age, ALBI score, tumor size, tumor number, or AFP between the two groups.

### 3.11 RFS after conversion therapy with curative or other therapies

**FIGURE 4:**
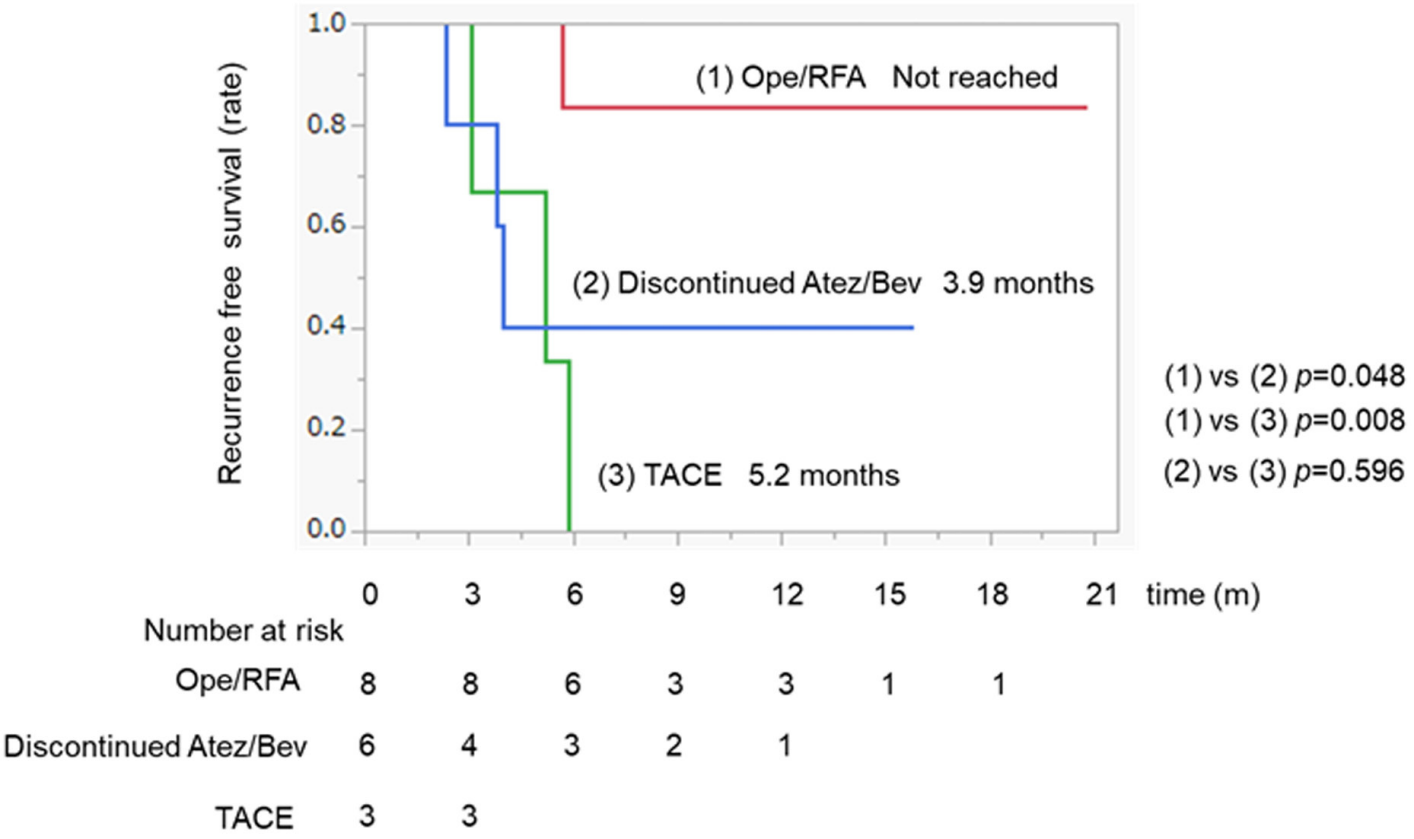
Recurrence‐free survival time after conversion therapy with curative or other therapies. The red, blue, and green line indicates the curative therapy, Atez/Bev discontinuation, and TACE group, respectively. Atez/Bev, atezolizumab plus bevacizumab; TACE, transarterial chemoembolization.

Figure 4 shows RFS after curative therapy, TACE, and Atez/Bev discontinuation. The curative therapy group had a significantly higher RFS than that in the other therapy groups.

### 3.12 AEs in the Atez/Bev treatment

Table S4 summarizes the Atez/Bev‐related AEs. The most common AE was hypertension, which occurred in 44.6% of the patients. Liver injury, fatigue, and proteinuria occurred in 41.0, 27.6%, and 26.3% of the patients, respectively.

## 4 DISCUSSION

In this multicenter investigation, we demonstrated that NLR <3 and m‐ALBI grade 1 or 2a were significant predictive factors for therapeutic response and conversion therapy, leading to cancer‐ and drug‐free patients treated with Atez/Bev. Moreover, we found that curative conversion therapy may be important in achieving long‐term RFS after Atez/Bev significant response.

We revealed that NLR <3 and m‐ALBI grade 1 or 2a were significant predictive factors for therapeutic response and conversion therapy leading to cancer‐ and drug‐free status in u‐HCC patients treated with Atez/Bev. Ochi et al. reported that low NLR can predict therapeutic response in patients with m‐ALBI grade 1 or 2a treated with Atez/Bev.^20^ Moreover, several studies have reported that NLR can predict outcomes in patients with u‐HCC treated with Atez/Bev, and these results are similar to those of previous studies that revealed that low NLR was associated with therapeutic response compared to high NLR.^21^, ^32^ NLR high is neutrophilia, which is related to high tumor‐associated macrophage infiltration and inflammatory cytokine production.^33^ In addition, relative lymphopenia impairs the host immune response against tumor cells, making it a poor prognostic factor for cancer patients.^34^ NLR is expected to serve as a baseline biomarker for patients receiving Atez/Bev because it is a simple biomarker for predicting inflammation in the body.^35^

Conversion therapy aims to reduce tumor volume and provide a chance at curative surgery for unresectable cancer patients.^36^ This concept is an important treatment strategy for several solid tumors, including colorectal cancer, gastric cancer, and pancreatic cancer. Various therapies are candidates for conversion therapy^37^, ^38^; however, this concept has never been developed because there is no evidence of treatment with a high therapeutic response for HCC. However, conversion therapy could be made possible for HCC through advances in systemic therapies. Several studies have reported that patients with u‐HCC could undergo hepatectomy after systemic therapy with combined tyrosine kinase inhibitor/anti‐PD‐1 antibodies.^39^, ^40^ Moreover, a previous study reported a significantly higher therapeutic effect among patients receiving Atez/Bev as the first‐line therapy than that in patients receiving Atez/Bev later.^41^ Although this study included approximately 40% of patients who were treated with Atez/Bev as the second‐line or later treatment strategies, the proportion of patients who were eligible for conversion therapy was 10.9%. In other words, as more cases were treated with Atez/Bev as the first line, the proportion of patients eligible for conversion therapy may increase. In this study, we also revealed that NLR <3 was associated with conversion therapy, leading to cancer‐ and drug‐free states. However, the OR rate was 16.3% in patients with NLR ≥3 by the decision tree. This response rate was higher than other systemic therapies. Therefore, we should not avoid patients from Atezo/Bev therapy group even without favorable factors. However, not all patients who achieved an OR are candidates for conversion therapy. Among the patients who had achieved OR, there were many cases wherein conversion therapy for a cancer‐free state could not be performed because of tumor and host factors, such as the number of tumors, tumor diameter, extrahepatic spread, vascular invasion, and deterioration of liver function. Moreover, the evidence of the clinical benefit of switching conversion therapy for patients receiving Atez/Bev is still not elucidated sufficiently. Thus, further study should focus on evaluating conversion therapy in u‐HCC patients treated with Atez/Bev.

In this study, our findings demonstrated that the curative therapy group had a significantly higher RFS than the non‐curative therapy group after Atez/Bev significant response. To our knowledge, this is the first study to show the importance of curative conversion therapy after Atez/Bev significant response. Recently, Xiao et al. reported that RFS was 75% at 12 months in HCC patients who underwent hepatectomy after treatment with tyrosine kinase inhibitor and anti‐PD‐1 Antibody combination therapy.^36^ In addition, several reports have demonstrated that disease‐free survival and OS were prolonged in patients who underwent liver resection or RFA as conversion therapy when tumors shrank after chemotherapy for colorectal cancer liver metastasis.^16^, ^17^, ^18^, ^19^ A previous study reported that the retreating liver metastasis leaves unresected, and more than half of patients with colorectal cancer will experience recurrence.^42^ These results suggest that the tumor should be completely removed whenever curative conversion therapy can be performed because the tiny cluster of viable tumor cells left could become a source of relapse, and predicting pathological CR before resection is impossible. Therefore, patients with tumor responses evaluated via images might benefit from curative therapy of residual lesions to achieve long‐term tumor‐free survival.

The present study had several limitations. First, it was a retrospective study. Second, the study included approximately 40% of patients who were treated with Atez/Bev as second‐line or later treatment. Third, HCC was not evaluated using The Liver Imaging Reporting and Data System.^43^ Fourth, a sufficient observation period for patients with curative conversion therapy after Atez/Bev significant response was not assessed. Thus, future evidence from prospective real‐world studies is needed to prove which conversion therapy is effective for patients with advanced HCC.

In conclusion, NLR <3 and m‐ALBI grade 1 or 2a were significant predictive factors for therapeutic response and conversion therapy, leading to cancer‐ and drug‐free patients with u‐HCC treated with Atez/Bev. Moreover, curative conversion therapy may be important in achieving long‐term RFS after Atez/Bev significant response.

### FUNDIND INFORMATION

This study has nothing to report.

## AUTHOR CONTRIBUTIONS

**Shigeo Shimose:** Conceptualization (lead); investigation (equal); project administration (equal); writing – original draft (lead). **Hideki Iwamoto:** Conceptualization (equal); formal analysis (equal). **Tomotake Shirono:** Data curation (supporting). **Masatoshi Tanaka:** Data curation (supporting). **Takashi Niizeki:** Data curation (equal). **Masahiko Kajiwara:** Data curation (equal). **Satoshi Itano:** Data curation (equal). **Yoichi Yano:** Data curation (equal). **Satoru Matsugaki:** Data curation (equal). **Etsuko Moriyama:** Data curation (equal). **Yu Noda:** Data curation (equal). **Masahito Nakano:** Data curation (equal). **Ryoko Kuromatsu:** Data curation (equal). **Hironori Koga:** Writing – review and editing (lead). **Takumi Kawaguchi:** Writing – review and editing (lead).

## FUNDIND INFORMATION

This study has nothing to report.

## CONFLICT OF INTEREST STATEMENT

Takumi Kawaguchi received honoraria (lecture fees) from Janssen Pharmaceutical K.K., Otsuka Pharmaceutical Co., Ltd, Taisho Pharmaceutical Co., Ltd., and EA Pharma Co., Ltd. The other authors declare no conflicts of interest.

## ETHICS APPROVAL AND CONSENT TO PARTICIPATE

The present study was approved by the Ethics Committee of Kurume University (approval code: 20183).

## Supporting information


Figure S1.

Figure S2.

Figure S3.

Table S1–S4.

## Data Availability

These data used and/or analyzed during the study are available from the corresponding author upon reasonable request.
