# Supplementary figures and images for: The impact of curative conversion therapy aimed at a cancer‐free state in patients with hepatocellular carcinoma treated with atezolizumab plus bevacizumab
Source: Cancer Med. 2023 Apr 16;12(11):12325–35. doi: 10.1002/cam4.5931 (PMC10278513; doi:10.1002/cam4.5931)

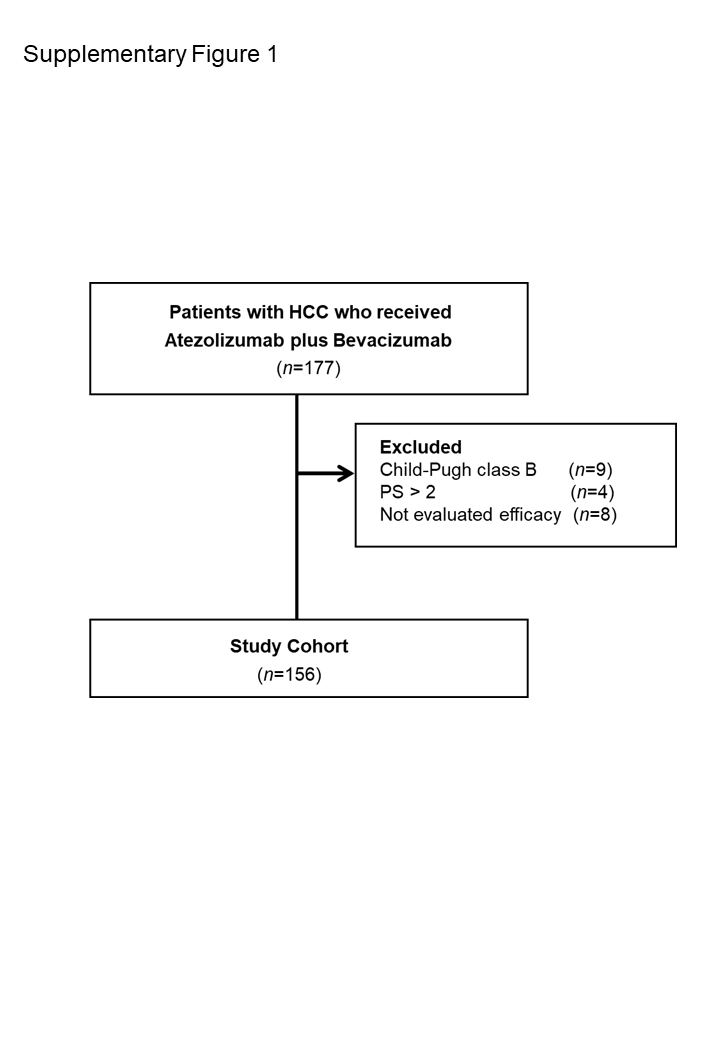

Supplement: Supplementary file 1 — Figure S1. [file CAM4-12-12325-s002.TIF]

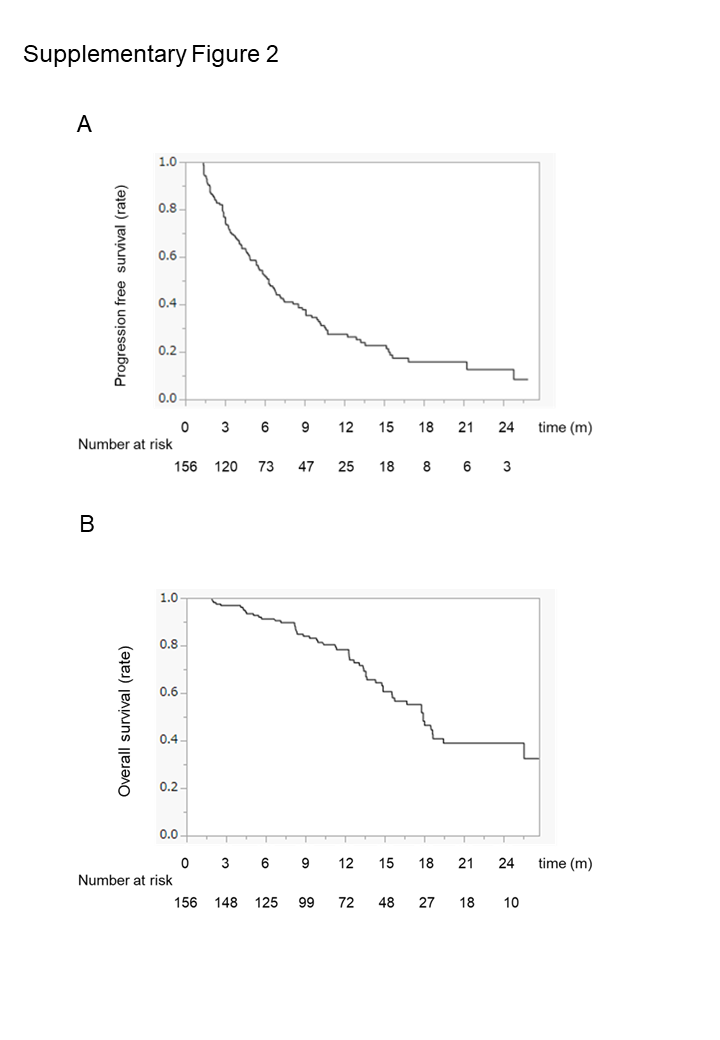

Supplement: Supplementary file 2 — Figure S2. [file CAM4-12-12325-s004.tif]

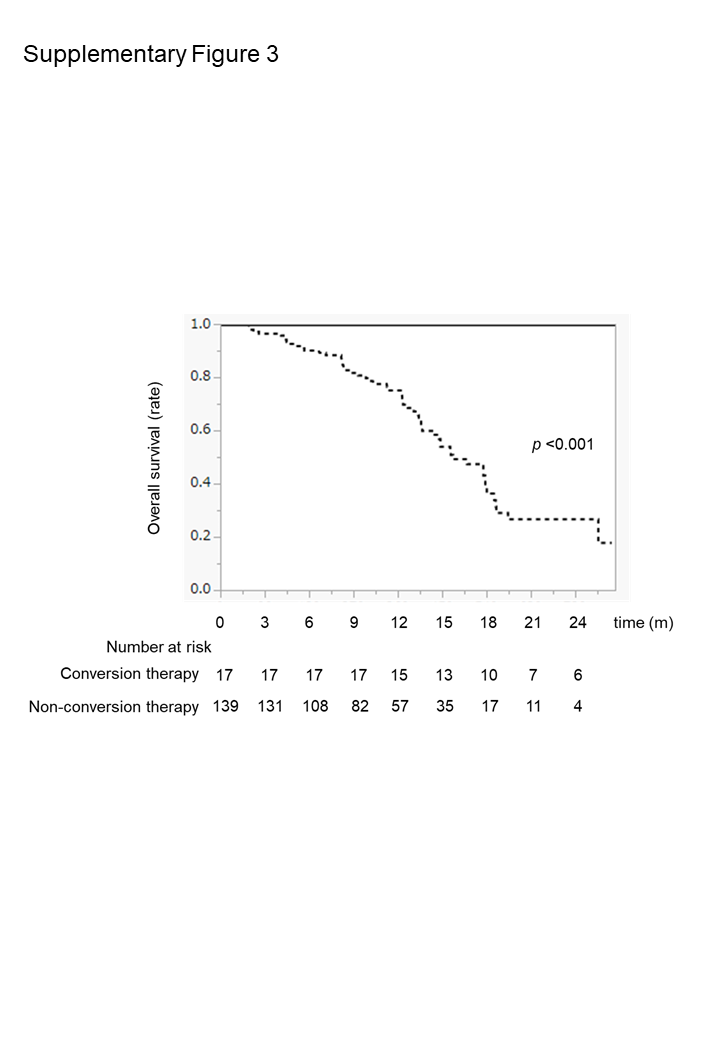

Supplement: Supplementary file 3 — Figure S3. [file CAM4-12-12325-s003.TIF]
