# Supplementary material for: The impact of curative conversion therapy aimed at a cancer‐free state in patients with hepatocellular carcinoma treated with atezolizumab plus bevacizumab
Source: Cancer Med. 2023 Apr 16;12(11):12325–35. doi: 10.1002/cam4.5931 (PMC10278513; doi:10.1002/cam4.5931)
Supplement: Supplementary file 4 — Table S1–S4. [file CAM4-12-12325-s001.docx]

Supplementary Table 1. Therapeutic responses according to RECIST (n=156)

| Best response |  |
| --- | --- |
| CR | 0 (0.0%) |
| PR | 50 (32.0%) |
| SD | 81 (52.0%) |
| PD | 25 (16.0%) |
| ORR | 50 (32.0%) |
| DCR | 131 (84.0%) |

Abbreviations: CR, complete response; PR, partial response; SD, stable disease; PD, progressive disease; ORR, objective response rate; DCR, disease control rate

Supplementary Table 2. Patient characteristics with NLR < 3 and ≥ 3

| Characteristic | NLR < 3  N=100 | NLR ≥ 3  N=56 | p |
| --- | --- | --- | --- |
| Age (years old) | 72 (37-93) | 74 (52-86) | 0.161 |
| Sex (female/male) | (22/78) | (13/43) | 0.861 |
| PS (0/1/2) | 83/17 | 45/11 | 0.679 |
| BMI | 23.0  (15.4-30.8) | 22.8  (16.6-35.2) | 0.745 |
| Etiology  (HBV/HCV/Non B, C) | (19/44/37) | (6/26/24) | 0.236 |
| ALBI score | -2.44  (-3.16– -1.55) | -2.39  (-3.50– -1.55) | 0.473 |
| m-ALBI grade (1/2a/2b) | (33/38/29) | (16/19/21) | 0.549 |
| BCLC stage (B/C) | (51/49) | (27/29) | 0.738 |
| Tumor diameter (mm) | 28.0 (10-136) | 41.5 (10-131) | 0.001 |
| Number of tumors  < 5/ ≥ 5 | (35/65) | (11/45) | 0.039 |
| Macrovascular invasion  (No/Yes) | 86/14 | 46/10 | 0.525 |
| Extrahepatic spread  (No/Yes) | 64/36 | 33/23 | 0.539 |
| AFP (ng/mL) | 35.6  (1.3-284,543) | 60.1  (1.2-279,663) | 0.456 |
| Treatment line  (1st/2nd/3rd/4th) | (62/31/4/3) | (33/18/4/1) | 0.081 |

Note. Data are expressed as median (range) or the number.

Abbreviations: NLR, neutrophil-to-lymphocyte ratio; PS, performance status; HBV, hepatitis B virus; HCV, hepatitis C virus; m-ALBI, modified Albumin-bilirubin grade; BCLC, Barcelona Clinic Liver Cancer; AFP, α-fetoprotein

Supplementary Table 3. Patient characteristics at the time of receiving conversion therapy with surgery or RFA and TACE

| Characteristic | Surgery or RFA  N=8 | TACE  N=3 | p |
| --- | --- | --- | --- |
| Age (years old) | 72 (55-86) | 83 (74-83) | 0.109 |
| Sex (female/male) | (2/6) | (2/1) | 0.200 |
| PS (0/1/2) | 7/1 | 3/0 | 0.520 |
| Etiology  (HBV/HCV/Non B, C) | (0/3/5) | (0/2/1) | 0.179 |
| ALBI score | -2.42  (-2.98– -1.91) | -2.50  (-2.73– -1.86) | 0.473 |
| Tumor diameter (mm) | 30.0 (12-81) | 27.0 (25-32) | 0.838 |
| Number of tumors  1  2  3 | 5  2  1 | 2  0  1 | 0.528 |
| AFP (ng/mL) | 19.5  (2.7-929) | 8.3  (6.4-141) | 0.918 |

Note. Data are expressed as median (range) or the number.

Abbreviations: RFA, radiofrequency ablation; TACE, transarterial chemoembolization; PS, performance status; HBV, hepatitis B virus; HCV, hepatitis C virus; ALBI, Albumin-bilirubin grade; AFP, α-fetoprotein

Supplementary Table 4. Adverse events associated with Atez/Bev (n=156)

| Adverse event | Any n (%) | Grade 3 ≥, n (%) |
| --- | --- | --- |
| Total adverse events | 149 (95.5%) | 60 (38.4%) |
| Hypertension | 68 (44.6%) | 14 (8.9%) |
| Liver injury | 64 (41.0%) | 8 (5.3%) |
| Fatigue | 43 (27.6%) | 2 (1.2%) |
| Proteinuria | 41 (26.3%) | 13 (8.3%) |
| Fever | 38 (24.3%) | 2 (1.2%) |
| Skin disorder | 37 (23.7%) | 2 (1.2%) |
| Appetite loss | 24 (15.4%) | 1 (0.6%) |
| Hoarseness | 23 (14.7%) | 0 (0.0%) |
| Hypothyroidism | 22 (14.1%) | 0 (0.0%) |
| Diarrhea | 19 (12.1%) | 1 (0.6%) |
| Bleeding | 18 (11.6%) | 10 (6.4%) |
| Hypopituitarism | 3 (2.3%) | 3 (2.3%) |
| Heart failure | 3 (2.3%) | 3 (2.3%) |
| Drug-induced pneumonia | 2 (1.2%) | 2 (1.5%) |
| Acute pancreatitis | 1 (0.6%) | 1 (0.6%) |
| Infusion reaction | 5 (3.2%) | 0 (0.0%) |

Abbreviations: Atez/Bev, atezolizumab plus bevacizumab
